# Implementation of automated behavior metrics to evaluate voluntary wheel running effects on inflammatory-erosive arthritis and interstitial lung disease in TNF-Tg mice

**DOI:** 10.1186/s13075-022-02985-6

**Published:** 2023-02-02

**Authors:** H. Mark Kenney, Ronald W. Wood, Gabriel Ramirez, Richard D. Bell, Kiana L. Chen, Lindsay Schnur, Homaira Rahimi, Benjamin D. Korman, Lianping Xing, Christopher T. Ritchlin, Edward M. Schwarz, Calvin L. Cole

**Affiliations:** 1 Center for Musculoskeletal Research, University of Rochester Medical Center, Rochester, NY USA; 2 Department of Pathology & Laboratory Medicine, University of Rochester Medical Center, Rochester, NY USA; 3 Department of Obstetrics and Gynecology, University of Rochester Medical Center, Rochester, NY USA; 4 Department of Neuroscience, University of Rochester Medical Center, Rochester, NY USA; 5 Department of Urology, University of Rochester Medical Center, Rochester, NY USA; 6 Department of Research, Hospital for Special Surgery, New York, NY USA; 7 Department of Pediatrics, Pediatric Rheumatology, University of Rochester Medical Center, Rochester, NY USA; 8 Department of Medicine, Division of Allergy, Immunology, Rheumatology, University of Rochester Medical Center, Rochester, NY USA; 9 Department of Orthopaedics, University of Rochester Medical Center, Rochester, NY USA; 10 Department of Surgery, University of Rochester Medical Center, Rochester, NY USA

**Keywords:** Mouse model, Arthritis, Lung, Micro-CT, Inflammation, Running, Exercise

## Abstract

**Background:**

Although treatment options and algorithms for rheumatoid arthritis (RA) have improved remarkably in recent decades, there continues to be no definitive cure or pharmacologic intervention with reliable long-term efficacy. For this reason, the combination of medications and healthy lifestyle modifications are essential for controlling joint disease, and extra-articular manifestations of RA, such as interstitial lung disease (ILD) and other lung pathologies, which greatly impact morbidity and mortality. Generally, exercise has been deemed beneficial in RA patients, and both patients and clinicians are motivated to incorporate effective non-pharmacologic interventions. However, there are limited evidence-based and specific exercise regimens available to support engagement in such activities for RA patients. Here, we provided the continuous opportunity for exercise to mice and implemented automated recording and quantification of wheel running behavior. This allowed us to describe the associated effects on the progression of inflammatory-erosive arthritis and ILD in the tumor necrosis factor transgenic (TNF-Tg) mouse model of RA.

**Methods:**

Wild-type (WT; males, *n*=9; females, *n*=9) and TNF-Tg (males, *n*=12; females, *n*=14) mice were singly housed with free access to a running wheel starting at 2 months until 5 to 5.5 months of age. Measures of running included distance, rate, length, and number of run bouts, which were derived from continuously recorded data streams collected automatically and in real-time. In vivo lung, ankle, and knee micro-computed tomography (micro-CT), along with terminal micro-CT and histology were performed to examine the association of running behaviors and disease progression relative to sedentary controls.

**Results:**

TNF-Tg males and females exhibited significantly reduced running distance, rate, length, and number of run bouts compared to WT counterparts by 5 months of age (*p*<0.0001). Compared to sedentary controls, running males and females showed increased aerated lung volumes (*p*<0.05) that were positively correlated with running distance and rate in female mice (WT: Distance, *ρ*=0.705/rate, *ρ*=0.693 (*p*<0.01); TNF-Tg: *ρ*=0.380 (*p*=0.06)/*ρ*=0.403 (*p*<0.05)). Talus bone volumes were significantly reduced in running versus sedentary males and negatively correlated with running distance and rate in TNF-Tg mice (male: *ρ*=−903/*ρ*=−0.865; female: *ρ*=−0.614/*ρ*=−0.594 (*p*<0.001)). Histopathology validated the lung and ankle micro-CT findings.

**Conclusions:**

Implementation of automated wheel running behavior metrics allows for evaluation of longitudinal activity modifications hands-off and in real-time to relate with biomarkers of disease severity. Through such analysis, we determined that wheel running activity increases aerated lung volumes, but exacerbates inflammatory-erosive arthritis in TNF-Tg mice. To the end of a clinically relevant model, additional functional assessment of these outcomes and studies of pain behavior are warranted.

**Supplementary Information:**

The online version contains supplementary material available at 10.1186/s13075-022-02985-6.

## Background

Rheumatoid arthritis (RA) is a chronic inflammatory disease characterized by pain, swelling, and decreased mobility of small joints affecting 0.5–1% of the worldwide population [1]. RA patients also suffer from extra-articular manifestations of the disease, which are important causes of morbidity and mortality [2], and ultimately contribute to the lower overall survival rate of RA patients [2–4]. Prominent among these is interstitial lung disease (ILD), which causes significant mortality [5, 6]. While formal algorithms for the management of RA patients exist [7, 8], there is no cure. Thus, efforts to incorporate healthful exercise in concert with prescribed immunosuppressive therapies have long been a goal of chronic RA therapy. However, the risk:benefit ratio of physical activity on inflamed joints remains poorly understood. There is continued controversy in the literature regarding the beneficial or detrimental effects of exercise in RA patients with the most recent clinical studies supporting the long-term benefits [9–12].

In an effort to better understand the effects of exercise on inflammatory-erosive arthritis in animals, investigators utilized voluntary wheel running and showed it to be sufficient to exacerbate surface erosions localized to entheses as opposed to articular surfaces in both acute (collagen-induced arthritis (CIA)) [13, 14] and chronic (tumor necrosis factor with AU-rich element deletion for systemic overexpression (TNF^ΔARE^)) [15] murine models of RA [16]. However, these outcomes were not directly correlated or attributed to running behaviors [17]. To address this important experimental limitation, we implemented automated continuous recording of wheel running to quantify micro-analytic metrics of activity, including running distance, rate, length, and number of bouts [18]. We subsequently correlated these behavioral endpoints with imaging biomarkers of arthritis and ILD.

In our previous studies, we utilized the TNF transgenic (TNF-Tg) mouse model of seronegative RA [19], which also displays ILD [20, 21]. Remarkably, TNF-Tg mice recapitulate the sexual dimorphisms of RA [22], as females exhibit accelerated onset of arthritis, ILD, and mortality [23]. Anti-TNF therapy has shown effectiveness in preventing and reducing the progression of these pathologies in TNF-Tg mice, but non-pharmacologic interventions have not yet been assessed [20, 24, 25]. As the natural history of disease in this well-established model of RA has been extensively described, we utilized these known pathologies to assess the predictive value of wheel running behavior to gain insights into the effects of exercise on the progression of arthritis and ILD in preclinical models.

## Methods

### Mouse models

All animal experiments were performed on IACUC-approved protocols through the University Committee for Animal Resources at the University of Rochester. TNF-Tg mice (3647 line) [15, 26, 27] were initially acquired from Dr. George Kollias and have since been maintained at the University of Rochester. The TNF-Tg mice were bred as heterozygotes, and wild-type (WT) littermates were used as controls (C57BL/6 genetic background). At 2 months of age, WT and TNF-Tg littermates were transferred from standard housing into single animal housing with an enriched environment containing continuous access to a running wheel. While sedentary measures for lung micro-CT were derived from our previously published historical data by Bell et al. [23] (permissions for reuse are described in Ethical Declarations, Consent for Publication), additional sedentary mice were placed in standard housing for ankle or knee micro-CT outcomes. To supplement animal availability, a non-functional loxP site was present in the platelet-derived growth factor B gene in a proportion of the mice ([28], Jackson Laboratory #017622), as part of a breeding regimen for a separate study; there were no notable functional differences in these mice. All mice were retained in a wheel-enriched environment until 5 to 5.5 months of age, estimating the median age of survival for TNF-Tg females [23], when they were euthanized for tissue collection. Disease-related outcome measures were evaluated by in vivo lung, ankle, and/or knee micro-computed tomography (micro-CT), and further assessed by terminal histology of the lungs and ankle joints. As female TNF-Tg mice exhibit an accelerated disease course [23], the females were measured at more frequent monthly intervals, while males were imaged at baseline and terminally. One WT female died unexpectedly due to technical error with positioning for micro-CT imaging, and five TNF-Tg females were unexpectedly found dead in their cages related to their disease course prior to scheduled euthanasia. A total of 80 mice were used in this study (running: *n*=42 mice; sedentary: *n*=38 mice), along with data from *n*=21 sedentary mice in Bell et al. [23]. The sample sizes for each group and outcome measure are provided in Supplementary Tables 1 and 2.

### Wheel running measurement

Mice were singly housed in a room on a 12:12 light cycle with the dark cycle beginning at 12:00AM to provide data synchronized to calendar days; this also enabled animal husbandry services during normal working hours. Mice were provided with free access to food and water as well as supplementary food enrichment (2-ounce cup of NutraGel Complete Nutrition, Bio-Serv) in small mouse cages (Tecniplast 1284L with lid and autoclavable filter top; West Chester PA). The dimensions of the cage were 365 × 207 × 140 mm with a floor area: 530 cm^2^; pilot work demonstrated that using larger cages resulted in delay or complete absence in the onset of stable wheel running patterns. An 11.5-cm diameter running wheel (Starr Life Sciences, Oakmont PA) was attached to the lid at one end of the cage adjacent to food and water bins; for further enrichment, a polycarbonate device was adjacent to the wheel (Safe Harbor Mouse Retreat; Bio-Serv, Flemington NJ), and a pad of compressed bedding (Nestlet; Ancare, Bellmore NY) was provided at each cage change. A neodymium magnetic disk attached to a wheel spoke closed a reed switch with each revolution. The reed switches were passed through a puncture in the filter top and connected to a digital interface (USB 6501, National Instruments, Austin TX) of a workstation on wheels equipped with an uninterruptable power supply so that recording could continue 24/7 without interruption.

### Wheel running analysis

LabVIEW (National Instruments, Austin TX) was used to automate all aspects of this experiment, and the software is available upon appropriate request from the University of Rochester. The digital interfaces were polled every 10 ms, a sampling rate adequate to ensure detection of switch closures at the most rapid revolution rate. Each switch closure resulted in the buffered recording of the timestamp; a new folder was created at midnight for the next day’s data.

To accommodate multiple experiments simultaneously, a wheel utilization manager was written that was used to assign (or release) a wheel to a user, and to record the cage card barcode, ear tag or other form of animal identification, date of birth, an experiment code, and a sex/transgene identifier. A diagnostic program was written to verify all wheels were working after weekly cage maintenance (i.e., provision of food and water). Cage bottoms were changed at least every 2 weeks under an IACUC-approved standard operating procedure; biweekly monitoring ammonia level (reflecting urine and fecal cage load) and ATP swabs for bacterial contamination and finding ammonia levels did not exceed 25 ppm and the wire lid swabs did not exceed 20,000 ATP units.

The time stamps were used to generate a behavioral micro-analysis of running behavior as described by Tepper et al. [18]. Wheel running is broken into runs or bouts by a pause (an interval between switch closures) of 9 s; this criterion interval was used to identify the number of runs (bouts per day), run length (revolutions (rev) per bout), running rate (rev per seconds), and distance per run (meters per bout). Number of runs per day was recorded as a marker of the tendency towards initiating this high probability behavior, which is reinforcing (i.e., rodents will press a lever to unlock a wheel and thus have an opportunity to run) [29]. As every switch closure is recorded as a time stamp, complete diurnal patterns of running were routinely examined and remained locked to the light cycle.

**Fig. 1:**
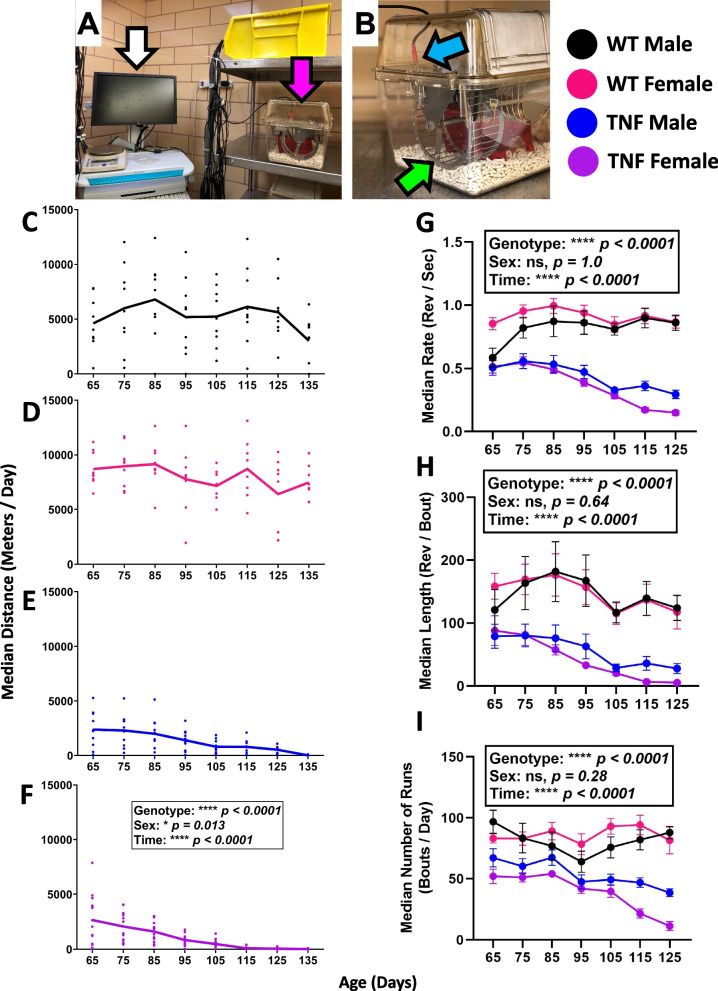
Automated wheel running metrics reveal a progressive decline in running performance for TNF-Tg mice. Wheel-enriched cages (pink arrow) were placed adjacent to a workstation (white arrow) with USB compatibility (**A**). A reed switch (blue arrow) wired to the workstation interface passed through a small gap in the cage lid, and securely attached to the base of the wheel. The wheel (green arrow) had a neodymium magnet attached to the outside rim facing the reed switch, such that each rotation of the wheel closed the reed switch (**B**). At 2 months of age, WT and TNF-Tg mice were placed individually in this enriched housing environment and provided free access to the running wheel. Daily activity metrics were collected and then transformed into 10-day medians synchronized to the date of birth. Median distance plots are provided for all groups (**C**–**F**) and showed running distance is dependent on genotype, sex, and time. In addition, derived metrics such as median running rate (**G**), running bout length (**H**), and number of runs (**I**) were also dependent on genotype and time, while sex was non-contributory. Statistics: 3-way mixed-effects; *p<0.05, ****p<0.0001

Running data was analyzed by 10-day medians for the outcome measures presented in Fig. 1 to depict and analyze running behavior over time with exclusion of day-to-day variation. The day-to-day variation tended to be either physiologic (i.e., fluctuating days of high and low activity, akin to “rest days” in exercise performance) or experimental (i.e., reduced access to the wheel due to imaging and associated recovery from anesthesia or cage changes). The running data is shown from age 65–135 days of age; initial datapoints at age 55 days were excluded due to variable initiation of running behavior, while any final day ranges after 5 months of age (>135 days) were excluded due to a range of euthanasia times for endpoints between 5 and 5.5 months of age. For derived measures (rate, length, number of runs), the final timepoint is at 125 days as running was absent in most TNF-Tg females afterwards. Sample sizes at each reported 10-day interval are described in Supplementary Table 1.

### Micro-CT analysis

Mice were anesthetized with 1–2% isoflurane for in vivo imaging of the lungs and joints using a VivaCT 40 (Scanco Medical, Bassersdorf, Switzerland). The following imaging parameters were used for the lung imaging: 45kV, 177μA, 120ms integration time, 1024 × 1024 pixels, 500 projections over 180°, and resolution of 35.0μm isotropic voxels. The imaging was gated to respiration, and thus images were only collected after passive exhalation (i.e., during the time between inspirations). DICOM files were imported into Amira software (v2020.2, ThermoFisher Scientific, FEI, Hillsboro, OR, USA), and the aerated, tissue, and total volumes were analyzed as previously described [30]. The female mice were evaluated at 3, 4, and 5.5 months of age, while male mice were assessed at 5.5 months of age to compare with sedentary data derived from our previously published study [23].

For the ankle and knee joints, the following imaging parameters were used: 55kV, 145μA, 300ms integration time, 2048 × 2048 pixels, 1000 projections over 180°, resolution 17.5μm isotropic voxels. Similarly, the DICOM files for the ankle datasets were imported into Amira, and the talus bone was used as a biomarker for the severity of inflammatory-erosive arthritis [25, 31], analyzed using previously described semi-automated segmentation methods [32]. The female mice were evaluated at 2, 3, 4, and 5 months of age, while male mice were assessed at 2 and 5 months of age. Ankle data from one sedentary WT female (3 months of age) and one running TNF-Tg female (3 months of age) were excluded due to motion artifact during imaging that did not allow for reliable analysis.

The knee micro-CT data was analyzed by snap-fit manual segmentation of the subchondral bone in the femur to derive trabecular outcome measures in the Scanco analysis software, including bone volume relative to total volume (BV/TV), trabecular number (Tb.N), trabecular thickness (Tb.Th), and trabecular separation (Tb.Sp), satisfying ASBMR reporting guidelines [33]. Specifically, femur subchondral analysis was performed by starting at the knee and proceeding proximally until the cortical shell was penetrated. Contours were closely drawn to the cortical shell and then shrunk to 95% in X and Y dimensions to avoid any inclusion of cortical bone. All non-cortical bone was included, both solid and trabecular. Proceeding proximally, all bone was included until the growth plate is reached. The growth plate was excluded. A Scanco threshold of 290 (2.320 cm^−1^, 3333 Hounsfield units) was used. Both female and male mice were evaluated at 2 and 5 months of age as measures of systemic osteoporotic bone loss did not reveal sexual dimorphic changes in previous studies of sedentary TNF-Tg mice by 5–6 months old [23]. For the ankle and knee micro-CT, individual limbs were used as the experimental unit given the well-established asymmetry of TNF-Tg inflammatory-erosive arthritis [23–25, 31, 34–36]. Sample sizes for micro-CT outcome measures are described in Supplementary Table 2.

### Tissue dissection and histology

Mice were euthanized with a cocktail of ketamine/xylazine followed by cardiac puncture. For the lung extraction, the trachea was identified and ligated with a single suture, then 10% neutral buffered formalin (NBF) was injected into the distal aspect of the trachea to fix the lung in an inflated position. The inflated lung was then further fixed in 10% NBF for 3 days and processed for paraffin-embedded histology with sections collected at 5μm across 3 tissue levels. A section from each level was then stained with hematoxylin and eosin (H&E) and imaged using an Olympus VS120 Slide Scanner. Lung sections from sedentary mice were derived from our historical data [23].

The ankle joints were also isolated, and surrounding soft tissue removed. The ankles were fixed for 3 days in 10% NBF and then decalcified in Webb-Jee 14% EDTA solution for 1 week. The joints were then processed for paraffin embedding to produce 5μm tissue sections across 3 tissue levels. A section from each level was then stained with H&E-Orange G (H&E-OG) for improved identification of bone tissue, and each section was imaged on an Olympus VS120 Slide Scanner. For both lung (total tissue and peri-arteriolar cell density) and ankle joint (peri-talar synovial infiltrate) histology, at least *n* = 3 mice were evaluated, and representative images are shown for qualitative comparison to exhibit the types of tissue changes associated with the quantitative micro-CT outcomes.

### Statistics

Two-way ANOVA and two/three-way mixed-effects statistical analyses were performed in GraphPad Prism (v9.4.1, San Diego, CA, USA), and the findings from these analyses are summarized in Table 1. Change in volume measurements was performed by evaluating percentage change from a 2-month baseline for the joints. Timepoints for cessation in total running distance were determined by one-sample *t*-tests comparing total distance to a value of zero, and the first timepoint to show no significant difference was defined as cessation in running activity.

**Table 1:**
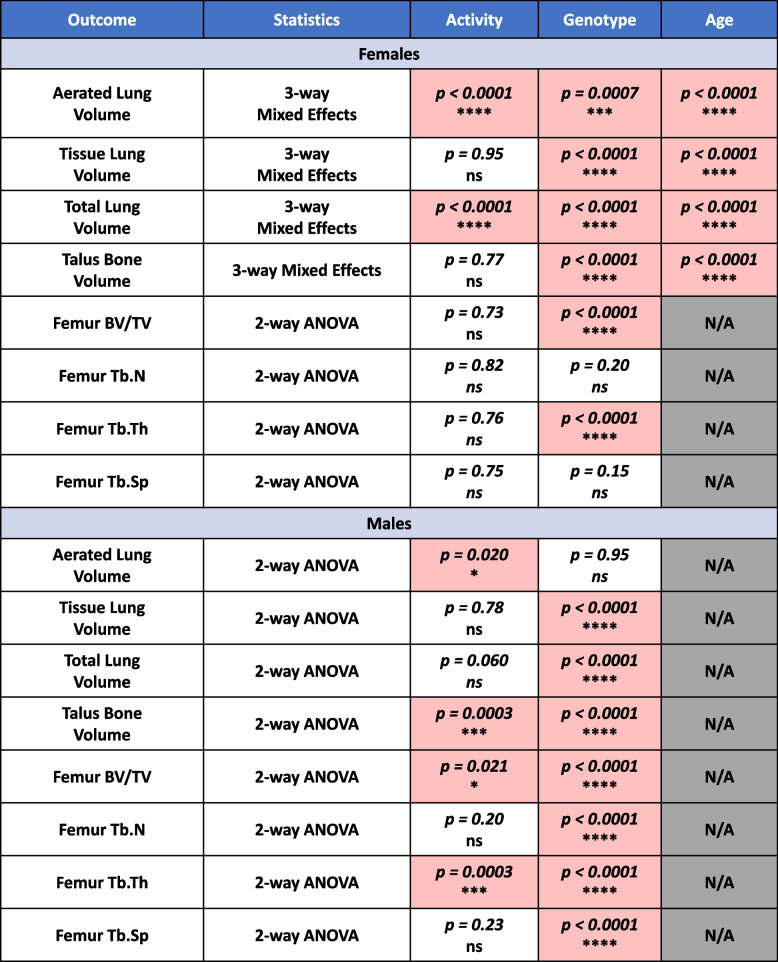
Statistical outcomes of micro-CT biomarkers for interstitial lung disease and inflammatory-erosive arthritis

**Fig. 2:**
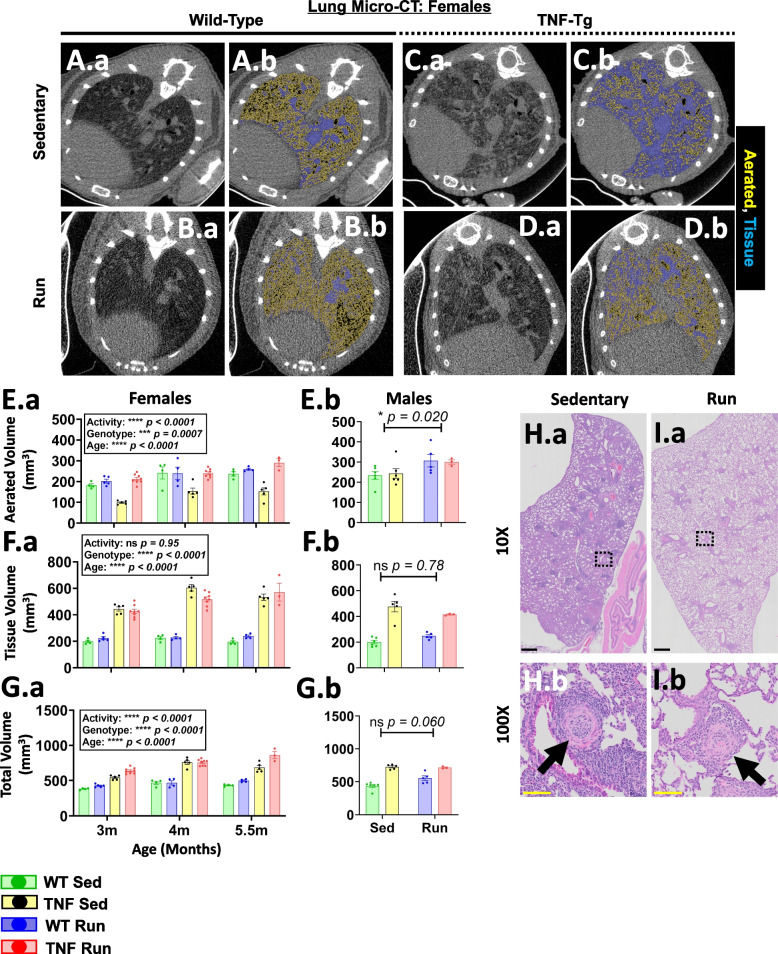
Voluntary wheel running enhances lung aeration in TNF-Tg mice without change in tissue volume. A representative cross-sectional image of a lung micro-CT dataset is provided for all groups of TNF-Tg female mice at 5.5 months of age (**A.a–D.a**) with associated segmentations of aerated (yellow) and tissue (blue) regions of the lung (**A.b–D.b**). Aerated volumes showed a significant dependence on activity and were increased for both females (**E.a**) and males (**E.b**), while tissue volumes were unaffected by activity (**F.a, F.b**). The increased aerated volume with activity similarly led to increased total lung volumes (**G.a, G.b**). Representative H&E-stained lung images from sedentary and running TNF-Tg females are provided, which demonstrates the relative increase in the proportion of aerated (white) to tissue (pink/purple) lung area for running TNF-Tg females with localized reductions in cellular infiltrate adjacent to arterioles (black arrows, **H.a–I.b**). Statistics: 3-way mixed-effects (**E.a–G.a**) and 2-way ANOVA (**E.b–G.b**); *p<0.05, ***p<0.001, ****p<0.0001. Black scale bar = 500μm (**H, I.a**), yellow scale bar = 100μm (**H, I.b**)

**Fig. 3:**
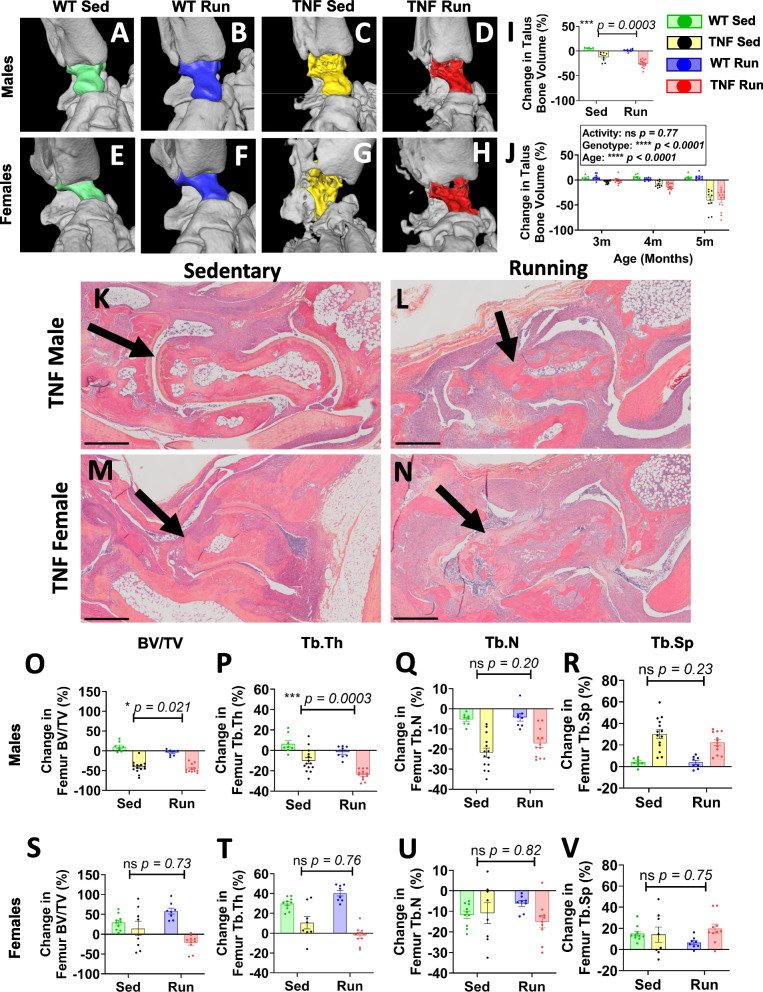
Inflammatory-erosive arthritis and generalized osteopenia are exacerbated by voluntary wheel running in TNF-Tg mice. Representative images of ankle joints by micro-CT are provided for each group, with the talus identified by color (**A**–**H**). Talus bone volumes were significantly dependent on and reduced with activity in male mice (**I**), but activity demonstrated no effect on female talus bone volumes **(J)**. Qualitative assessment of representative H&E-OG stained ankle joints showed a remarkable increase in synovial inflammation (purple) surrounding the talus (arrows) with running activity for both male (**K**, **L**) and female (**M**, **N**) TNF-Tg mice. In addition, subchondral bone metrics in the femur (bone volume per total volume = BV/TV, trabecular thickness = Tb.Th, trabecular number = Tb.N, trabecular separation = Tb.Sp) demonstrated reduced BV/TV and Tb.Th with running activity in male mice (**O**–**R**). On the other hand, female mice (**S**–**V**) showed no change in subchondral bone metrics related to activity. Statistics: 3-way mixed-effects (**J**) and 2-way ANOVA (**I**, **O**–**V**); *p<0.05, ***p<0.001, ****p<0.0001. Black scale bars = 500μm (**K**–**N**)

The statistical outcomes for the interaction analyses of the lung (Fig. 2; females: 3-way mixed effects/males: 2-way ANOVA), talus (Fig. 3; females: 3-way mixed effects/males: 2-way ANOVA), and subchondral femur (Fig. 3; 2-way ANOVA) volume metrics are provided in tabular format. Significant effects (*p*<0.05) are shown in red

For correlative outcomes, the running metrics were logarithmically transformed to meet the normality assumptions of regression analysis. Normalization had the additional benefit of circumventing discretization of the continuous outcome and time variables allowing for increased statistical power. Several regression diagnostics were used to justify this normalization. The change in running outcome variables since the first observation was used in the regression models to facilitate post hoc contrasts. Association between TNF-Tg status, sex, and running outcome was estimated using a mixed effects regression model to account for the longitudinal aspect of the data. Correlations between micro-CT and running outcomes were performed using partial correlations based on a mixed effects regression model, using time as the only fixed effect, to account for the longitudinal aspect of the data. Regression analysis, post hoc contrasts, and partial correlations were performed using STATA v16.2 (STATA Corp., College Station, TX).

## Results

### Automated wheel running metrics reveal a progressive decline in running performance for TNF-Tg mice

To describe progressive changes in running behavior in TNF-Tg mice, and the association with the progression of ILD and inflammatory-erosive arthritis, mice were singly housed in an enriched environment with free access to a running wheel (Fig. 1A, B). Wheel revolutions were detected using running wheels with an attached neodymium magnet that closed a reed switch with each revolution, enabling automated collection of measures, such as running distance, rate (rev/s), length (rev/bout), and number of runs (bouts/day). A bout was defined as successive switch closures less than 9 s apart. Both TNF-Tg males and females exhibited reduced daily running distance compared to their WT counterparts (genotype, *p*<0.0001) that progressed over time (time, *p*<0.0001) (WT males: start = 4604.7±2428.9/end = 3007.4±2216.6, WT females: 8703.3±1541.4/7472.8±1559.2, TNF-Tg males: 2380.4±1687.7/17.1±35.4, TNF-Tg females: 2660.1±2284.7/9.9±31.1 10-day median of meters/day). In addition, WT females demonstrated longer running distance compared to WT males, while TNF-Tg females stopped engaging in running behavior sooner compared to TNF-Tg males (TNF-Tg males 140 days vs TNF-Tg females 113 days to running cessation; sex, *p*<0.05) (Fig. 1C–F; black = WT male, pink = WT female, blue = TNF male, purple = TNF female). Both TNF-Tg males and females also showed a progressive reduction in running rate (WT male: start = 0.6±0.2/end = 0.9±0.2, WT female: 0.9±0.1/0.9±0.1, TNF-Tg male: 0.5±0.2/0.3±0.1, TNF-Tg female: 0.5±0.2/0.1±0.1 10-day median of rev/s), running length (WT male: start = 120.8±97.7/end = 124.0±59.8, WT female: 158.5±61.9/117.6±75.3, TNF-Tg male: 79.2±59.8/28.0±25.3, TNF-Tg female: 88.1±87.8/5.4±6.1 10-day median of rev/bout), and number of runs (WT male: start = 96.8±28.6/end = 87.9±15.4, WT females: 83.2±11.2/81.5±30.9, TNF-Tg males: 67.2±23.7/38.5±10.3, TNF-Tg females: 52.0±22.3/11.4±10.9 10-day median of bouts/day) compared to their WT counterparts (Fig. 1G–I; genotype and time, *p*<0.0001), but there was no interaction effect of sex on these running endpoints. To illustrate the dramatic genotype effects on running rate, representative videos of running WT and TNF-Tg females at 5 months of age are provided in SupplementaryVideos. As TNF-Tg females are known to exhibit remarkably accelerated ILD and inflammatory-erosive arthritis compared to males [23], the limited difference in running behavior between TNF-Tg males and females throughout the measurement period suggests that there may be common mechanisms regulating changes in activity beyond biomarkers of disease, such as pain.

### Voluntary wheel running enhances lung aeration in TNF-Tg mice without change in tissue volume

As we demonstrated previously that TNF-Tg mice exhibit ILD with remarkably increased disease severity and associated mortality in females [20, 23], we sought to determine whether free access to a running wheel could alter pulmonary outcomes. In vivo micro-CT volume measurements correlate with outcomes of pulmonary function tests in TNF-Tg mice [37], and thus we evaluated aerated, tissue, and total lung volumes via established analytical protocols [30]. Representative cross-sectional lung micro-CT images are shown for all groups and demonstrate a dramatic increase in aerated lung regions (yellow) for running TNF-Tg cohorts compared to sedentary controls (females: Fig. 2A.a–D.b, Males: Supplementary Figure 1). In fact, both female and male mice exhibited significantly increased aerated lung volume with activity (5.5-month sedentary vs. running, respectively for all groups; WT female: = 236.8±21.1/259±10.1, TNF-Tg female: 152.5±38.2/290.7±32.8, WT male: 233.8±46.3/306.5±67.8, TNF-Tg male: 243.2±58.3/300.3±19.0 mm^3^ aerated lung volume; Fig. 2E.a,b, Activity *p*<0.05 both sexes). However, tissue volume, a biomarker of healthy interstitium plus pathologic cellular infiltrate, was unchanged with running behavior (5.5-month sedentary vs. running, respectively for all groups; WT female: 195.4± 19.1/238.9± 17.8, TNF-Tg female: 532.8± 53.6/571.5± 116.6, WT male: 200.2± 33.8/247.5± 24.7, TNF-Tg male: 476.3± 91.1/414.9± 7.3 mm^3^ tissue lung volume; Fig. 2F.a,b, Activity *p*>0.05 both sexes). Together, these changes translated to increased total lung volumes with activity (5.5-month sedentary vs. running, respectively for all groups; WT female: 432.1±15.1/498.2±20.8; TNF-Tg female: 685.3±66.1/862.2±89.4, WT male: 434.0±57.3/554.0±79.5, TNF-Tg male: 725.5±36.5/715.2±17.4 mm^3^ total lung volume; Fig. 2G.a,b, Activity *p*<0.0001 females, *p*=0.06 males).

Remarkably, non-linear regression analysis revealed that the running distance and rate of female mice exhibited a positive correlation with aerated lung volumes (Table 2; distance, WT: *ρ*=0.705 (*p*<0.01), TNF-Tg: *ρ*=0.380 (*p*=0.06); rate, WT: *ρ*=0.693 (*p*<0.01), TNF-Tg: *ρ*=0.403 (*p*<0.05)), further demonstrating the integral relationship between running activity and micro-CT outcomes in the lung. However, running activity was unrelated to distinct changes in aerated lung volume in male mice (Table 2; distance, WT: *ρ*=0.412, TNF-Tg: *ρ*=0.677; rate, WT: *ρ*=0.379, TNF-Tg: *ρ*=0.647; *p*>0.05). Representative H&E-stained lung sections from sedentary and running TNF-Tg mice corroborated the micro-CT findings, which showed a relative decrease in the proportion of tissue area (females: Fig. 2H.a, I.a; males: Supplementary Figure 1, pink/purple relative to white regions). There was also a notable and localized reduction in cellular density surrounding the arterioles (females: Fig. 2H.b, I.b; males: Supplementary Figure 1, black arrows). Similar pathologic peri-arteriolar cellular infiltrate was previously described in TNF-Tg mice with severe ILD and pulmonary arterial hypertension, which may depict either inflammation or vascular remodeling [38]. These findings demonstrate that wheel running is sufficient to improve aerated lung volumes in TNF-Tg mice, and females with greater severity of ILD exhibit enhanced sensitivity to these effects that directly correlate with running behavior.

**Table 2:**
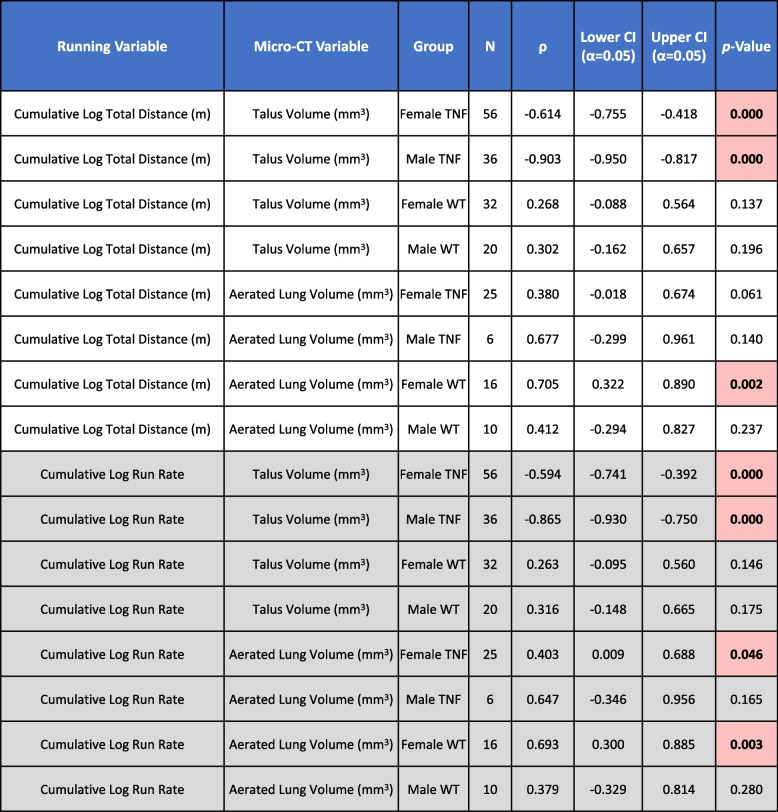
Running measures correlate with increased lung aeration and reduced bone volumes in TNF-Tg mice

We evaluated the direct effects of running distance and rate (Fig. 1) on the longitudinal micro-CT outcome measures of aerated lung (Fig. 2) and talus bone (Fig. 3) volumes by non-linear regression analysis. Total distance and running rate were significantly and negatively correlated with talus bone volumes for both female (distance: *ρ*=−0.614/rate: *ρ*=−0.594) and male (*ρ*=−0.903/*ρ*=−0.865) TNF-Tg mice (*p*<0.001). In addition, total distance and running rate were positively correlated with aerated lung volumes for female TNF-Tg (*ρ*=0.380 (*p*=0.061)/*ρ*=0.403 (*p*<0.05)) and WT (*ρ*=0.705/*ρ*=0.693 (*p*<0.01)) mice, but showed no relationship with males (*p*>0.05). Sample size is determined by number of limbs (talus) or animals (lung) at each time point evaluated. Significant effects (*p*<0.05) are shown in red

### Inflammatory-erosive arthritis and generalized osteopenia are exacerbated by voluntary wheel running in TNF-Tg mice

Cambré et al. previously demonstrated that acute CIA and chronic TNF^ΔARE^ mouse models exhibit increased surface erosions at enthesis sites with voluntary wheel running [17]. However, the bone pathology was notably variable, not stratified by sex, and not directly attributed to the degree of running behavior by regression analysis. Thus, we utilized semi-automated segmentation protocols to quantify the talus bone volume [32], a biomarker of inflammatory-erosive arthritis in TNF-Tg mice [31], and evaluated the changes in bone volume relative to running activity. Representative ankle micro-CT images are shown with the talus bone highlighted for each group; for the TNF-Tg mice, we observed severe bone erosions localized to the articular surfaces, most clearly exhibited by the reduced bone volume at the tibiotalar joint in the provided images (Fig. 3A–H). Male mice demonstrated a significant reduction in talus bone volumes related to running, while females showed no effect of activity on the severity of erosive arthritis (5-month sedentary vs. running, respectively for all groups; WT males: = 5.4±2.3/1.4±2.5, TNF-Tg males: −12.5±9.4/−25.5±7.1, WT females: 5.2±4.4/5.9±5.9, TNF-Tg females: −40.4±20.3/−39.4±20.3% change from 2-month baseline volume; Fig. 3I, J, activity *p*<0.001 males, *p*=0.77 females). Importantly, non-linear regression analysis revealed that talus bone volumes were negatively correlated with running distance and rate for both TNF-Tg males (Table 2; distance, WT: *ρ*=0.302 (*p*>0.05), TNF-Tg: *ρ*=−0.903 (*p*<0.01); rate, WT: *ρ*=0.316 (*p*>0.05), TNF-Tg: *ρ*=−0.865 (*p*<0.01)) and females (distance, WT: *ρ*=0.268 (*p*>0.05), TNF-Tg: *ρ*=−0.614 (*p*<0.01); rate, WT: *ρ*=0.263 (*p*>0.05), TNF-Tg: *ρ*=−0.594 (*p*<0.01)), which demonstrated that the degree of activity was directly linked with bone erosions (Table 2). Despite the lack of erosive changes noted by micro-CT in TNF-Tg female ankles with running compared to sedentary, both male and female TNF-Tg running cohorts exhibited dramatically increased inflammatory infiltrates (purple) surrounding the talus (black arrows) by H&E-OG staining (Fig. 3K–N).

We also evaluated the effects of activity on generalized osteopenia in the subchondral region of the femur, which has been demonstrated to exhibit a differential response to TNF compared to the articular surfaces of bone [39]. Similarly, activity led to reduced subchondral bone volume (BV/TV; WT males: sedentary = 10.2±11.8/running = −4.3±7.5, TNF-Tg males: −41.3±14.9/−45.1±10.4, WT females = 29.7±19.2/58.2±20.9, TNF-Tg females = 14.3±52.7/−21.2±19.8% change from 2-month baseline) and trabecular thickness (Tb.Th; WT males: sedentary = 6.5±9.0/running = −1.7±5.8, TNF-Tg males: −10.2±11.3/−23.8±5.1, WT females: 29.6±6.2/40.3±8.2, TNF-Tg females: 10.7±17.5/−2.2±9.1% change from 2-month baseline) in male mice, while female mice showed no changes in these bone metrics with activity. Both sexes exhibited no changes in trabecular number (Tb.N; WT males: sedentary = −5.2±2.8/running = −4.2±5.2, TNF-Tg males: −21.8±8.5/−17.1±7.2, WT females: −11.7±5.5/−6.2±3.9, TNF-Tg females: −10.9±14.4/−15.0±9.5% change from 2-month baseline) or separation (Tb.Sp; WT males: sedentary = 4.1±3.7/running = 3.8±5.5, TNF-Tg males: 30.2±15.0/22.3±9.9, WT females: 14.9±7.1/6.5±5.3, TNF-Tg females: 14.1±20.2/19.8±13.4% change from 2-month baseline) in response to activity (Fig. 3O–V). Together, these findings demonstrate that the severity of inflammatory-erosive arthritis and generalized bone loss are dependent on the degree of wheel running activity. Furthermore, wheel running exacerbates these pathologic features in TNF-Tg males, while females display a ceiling effect on measures of disease severity independent of activity by 5 months of age.

## Discussion

While exercise is broadly considered to be healthful to all animals, different types (or degree) of exercise activity may be contraindicated for individuals with injuries and chronic inflammatory diseases like RA. Thus, quantitative thresholds of exercise intensity and duration are needed to optimize the risk:benefit ratio for compromised individuals. Exercise endpoints also enhance preclinical research aimed at elucidating the mechanisms responsible for the benefits and harm caused by exercise in animal models. To this end, we implemented automated methods to record running behavior in unsupervised mice, which included running distance, rate, length, and run bouts, hands-off and in real-time. With this approach, we evaluated the association of running metrics on the severity of ILD and inflammatory-erosive arthritis in the TNF-Tg mouse model. We found that greater running distance and rate are sufficient to increase aerated lung volume in TNF-Tg mice, while also enhancing joint erosions and systemic bone loss in TNF-Tg males.

The sexual dimorphism in these responses may be explained by differential mechanisms in disease activity. For instance, although previous studies of TNF-Tg mice have noted limited sexual dimorphism in systemic bone loss [23], we unexpectedly discovered that running males exhibit an accelerated decrease in subchondral trabecular bone that does not occur in females. On the other hand, females instead show a trend towards subchondral bone maintenance with minimal loss regardless of activity. Of note, subchondral bone marrow changes in RA are thought to serve as a source of osteoclastogenesis, thus promoting local joint damage [40, 41]. Therefore, the exacerbated subchondral and focal joint erosions may be mechanistically related in male mice, but function through separate pathways in females. These findings support further investigation into the mechanisms that mediate the sexually dimorphic progression of joint disease in TNF-Tg mice, which may guide understanding into the similar sex-based differences noted in clinical RA.

Alternatively, the sexual dimorphic responses may be a product of the known accelerated severity in these pathologic processes for TNF-Tg females. For example, the direct association of running behaviors with increased aerated lung volumes in TNF-Tg females, but not males, may be related to a greater capacity for improvement in these micro-CT outcomes as females start at a baseline of more severe disease. Similarly, erosive arthritis and generalized bone loss may only be exacerbated in males because females have already reached a ceiling of disease activity where further damage from activity could not be detected via micro-CT volume measurements. For instance, in active inflammatory arthritis, erosive activity overwhelms bone remodeling [42], but as bone volume is reduced, pathologic bone formation (i.e., osteophyte growth) may eventually equalize with bone loss such that total volume changes are undetectable, although shape and function may continue to be altered. As such, pathologic bone formation is related to disease duration and extent of erosions in RA [43] and may explain the inability to detect bone volume changes in females with more severe arthritis at baseline. However, histologic assessment suggested that TNF-Tg mice, regardless of sex, exhibited increased inflammatory infiltrate adjacent to the talus bone, accumulating in the synovium independent of continued measurable bone loss, which may also negatively impact joint function and mobility.

At present, it remains questionable whether the results of this animal study have clinical relevance for human RA. Nevertheless, the preclinical findings in this work may indeed provide valuable precedents and justification for clinical trials to assess the impact of activity on RA progression. In fact, TNF-Tg mice (3647 line, single copy of human TNF) serve as a reliable seronegative model [19] and recapitulate many aspects of human disease. For instance, clinical RA is not only more prevalent in females compared to males, but females tend to also experience more severe erosive joint disease, lower rates of remission, and increased functional decline [44–47], similarly demonstrated in the TNF-Tg mice [23]. The TNF-Tg mice also exhibit a specific phenotype of ILD, nonspecific interstitial pneumonia (NSIP, inflammatory and fibrotic) [20], more prevalent clinically in females [48] relative to usual interstitial pneumonia (UIP, predominately fibrotic), which has a predilection to affect males [49]. The female predominance of NSIP RA-ILD clinically may, in part, explain the accelerated cardiopulmonary decline in TNF-Tg females compared to males, recapitulating the increased susceptibility of females to NSIP pathology. As the sexual dimorphism in TNF-Tg mice closely mirrors human RA, modulating disease activity through non-pharmacologic interventions in this preclinical model provides valuable opportunities to investigate the sex-based determinants of RA pathogenesis with potential for clinical translation. Through implementation of automated activity metrics, our findings and future work may utilize this robust preclinical system to further investigate the effects of exercise modalities on disease outcome measures, or vice versa, to inform clinical studies.

One of the primary limitations in our study is the lack of functional outcomes for arthritis and ILD. As we have demonstrated the remarkable impact of voluntary running on micro-CT biomarkers of these disease processes, this warrants investigation in future studies to evaluate the functional consequences of these changes. For instance, restrictive lung disease severity measured by pulmonary function tests showed a correlation with lung tissue volumes by micro-CT [37]. Further studies in running TNF-Tg mice could assess the pulmonary functional changes with activity and thus explain the potential benefits of increased aerated lung volumes, which conceptually equate to functional residual capacity, given that imaging sequences are collected at passive exhalation. The effects of running on the lung could also be evaluated in the context of cardiac disease as well, as severe and lethal pulmonary arterial hypertension and right ventricular hypertrophy is inherent in sedentary TNF-Tg mice and may be modified by exercise [38]. In addition, the functional effects of arthritic changes warrant assessment by gait analysis [50]. Exercise has been previously shown in injury models of osteoarthritis in rats to promote improved gait with reduced compensations [51], which may support functional recovery. On the other hand, it may be hypothesized that, in TNF-Tg mice, weight-bearing aerobic exercise (i.e., wheel running) may exacerbate gait disturbances to further accelerate disease progression by promoting non-ergonomic paw placements to alleviate the progression of pain that could also be evaluated preclinically. Such analysis would also benefit from the evaluation of skeletal muscle pathology on functional changes as well, since TNF-Tg mice are known to develop sarcopenia [52]. In addition, physical activity has been shown to improve muscle outcomes in chronic inflammatory conditions, potentially through reduced TNF activity [53, 54].

Based on the running data, it is important to note that both male and female mice dramatically reduce in activity within 5 months of age, at a time when cardiopulmonary disease in males is known to be relatively mild compared to females. As the exercise exacerbated inflammatory-erosive arthritis and generalized bone loss in male TNF-Tg mice, the reduction in running behavior is more likely related to these changes detrimental to bone and joint health. However, bone metrics in TNF-Tg females continued to be more severe than male counterparts, suggesting that our disease biomarkers are insufficient to fully explain the comparable changes in running behavior. It remains possible that unmeasured symptoms related to bone and joint disease, such as pain, may be the dominant feature that ultimately reduces activity in TNF-Tg mice and may eventually negate the early beneficial effects of wheel running on increasing aerated lung volumes. Our imaging evaluation was unable to evaluate the ways in which these structural changes may translate into symptomatic pain that can drive changes in behavior. Magnetic resonance imaging has demonstrated that bone marrow lesions in subchondral regions are an adequate biomarker to evaluate pain in osteoarthritis [55–57]. Utilization of such analytical techniques in future studies may aid in elucidating potential localized regions of pain associated with reduced activity towards guiding the development of mechanical bracing or pharmaceutical interventions to target the pain response.

There is also controversy in the literature regarding the beneficial or detrimental effects of exercise on the articular and extra-articular manifestations of inflammatory arthritis. Particularly in RA, early literature expressed concern that exercise may exacerbate joint disease, but more recent studies have supported long-term benefits on cardiovascular, joint, and overall function in RA patients [9–12]. However, similar to our findings, recent animal studies in mouse models of RA have suggested that voluntary exercise in the form of wheel running may indeed exacerbate joint inflammation and erosions [17]. The lack of consensus regarding the effects of exercise on RA progression has led to patient confusion, where the particular types of activity that may be beneficial remain unclear [58]. In fact, there are tremendous complications with the application of exercise regimens as each patient will have different levels of disease activity, depending on current pharmacologic treatments and their effectiveness. Future studies ought to consider purposeful stratification of disease activity and exercise approaches, such as aerobic (running vs swimming, etc.) or strength (weight vs non-weight bearing, etc.) training, along with consideration for timing (number of days, length of workout) and intensity, to provide more explicit exercise recommendations [11]. For instance, in this study we demonstrated that daily wheel running, an aerobic weight-bearing activity, indeed exacerbates arthritis in an untreated TNF-Tg model of RA, and arthritic severity is directly correlated with increased activity. Through collaboration with therapy providers, development of an evidence-based exercise program could remarkably improve patient care by maximizing the benefits of exercise, while minimizing potential detrimental effects on joint health.

## Conclusions

In this work, we have implemented automated quantification of wheel running metrics including distance and detailed characterization of running bouts. We demonstrated the utility of monitoring running in the TNF-Tg mouse model of RA through remarkable changes in running outcomes for TNF-Tg vs WT mice and the effects of exercise on ILD and inflammatory-arthritis severity. Specifically, increased wheel running activity is associated with increased aerated lung volume and exacerbation of arthritis, as measured by micro-CT endpoints. Through these findings, we demonstrated the utility of monitoring voluntary running in preclinical murine models and identified valuable disease-modifying effects of these activities that may help future research focused on the design of exercise strategies for RA patients.

## Supplementary Information

**Additional file 1: Supplementary Figure 1.** Depiction of lung micro-CT, segmentation, and histology in male mice. Representative cross-sectional image of lung micro-CT datasets (**A.a-D.a**) and associated segmentations of aerated (yellow) and tissue (blue) lung regions (**A.b-D.b**) are provided for each group of male mice. Representative H&E-stained lung images from TNF-Tg male sedentary versus running mice are shown, where little change in morphology was appreciated in the tissue as a whole or adjacent to the arterioles (black arrows) (**E.a-F.b**). Black scale bar = 500μm (**E,F.a**), yellow scale bar = 50μm (**E,F.b**). **Supplementary Table 1.** Sample sizes for running data. A table is provided with the sample sizes (number of animals) for each group and timepoint. For each group (WT male/female, TNF-Tg male/female), the sample sizes are shown at the following time points (days, outcomes in 10-day median blocks): 65 / 75 / 85 / 95 / 105 / 115 / 125 / 135. Reductions in sample size indicate mortality during the study period. **Supplementary Table 2.** Sample sizes for micro-CT outcome measures. A table is provided with the sample sizes for each group and timepoint. The groups include sedentary and running cohorts of WT male/female and TNF-Tg male/female mice. The joint micro-CT outcomes were measured as a change from a 2-month baseline. For the lung and ankle micro-CT, the males show 5 or 5.5-month measurements, while the females exhibit 3 / 4 / 5 or 5.5-month outcomes. The knee micro-CT represents outcomes at 5-month measures for both males and females. Changes in sample size across timepoints either represent exclusion of datasets due to motion artifact or animal mortality during the study period. ^a^Final timepoint represents 5.5-months of age and experiment unit is number of mice, ^b^final timepoint represents 5-months of age and experimental unit is number of limbs, *sedentary measurements are derived from historical data in our previous publication [23].

**Additional file 2: Supplementary Videos.** Real-time video of wheel running activity in a wild-type female at 5-months of age

**Additional file 3: Supplementary Videos.** Real-time video of wheel running activity in a TNF-Tg female at 5-months of age

## Data Availability

All datasets and materials will be made available upon reasonable request. Any requests regarding the data reproduced from Bell et al. [23] must abide by the permissions described below under Ethical Declarations, Consent for Publication.

## Abbreviations

RA: Rheumatoid arthritis
ILD: Interstitial lung disease
CIA: Collagen-induced arthritis
TNF: Tumor necrosis factor
ARE: AU-rich elements
TNF-Tg: TNF-transgenic
WT: Wild-type
Micro-CT: Micro-computed tomography
Rev: Revolutions
BV/TV: Bone volume per total volume
Tb.N: Trabecular number
Tb.Th: Trabecular thickness
Tb.Sp: Trabecular separation
NBF: Neutral buffered formalin
H&E: Hematoxylin and eosin
H&E-OG: H&E-Orange G
NSIP: Nonspecific interstitial pneumonia
UIP: Usual interstitial pneumonia
