# Supplementary material for: Implementation of automated behavior metrics to evaluate voluntary wheel running effects on inflammatory-erosive arthritis and interstitial lung disease in TNF-Tg mice
Source: Arthritis Res Ther. 2023 Feb 2;25:17. doi: 10.1186/s13075-022-02985-6 (PMC9893562; doi:10.1186/s13075-022-02985-6)
Supplement: Supplementary file 1 — Additional file 1: Supplementary Figure 1. Depiction of lung micro-CT, segmentation, and histology in male mice. Representative cross-sectional image of lung micro-CT datasets (A.a-D.a) and associated segmentations of aerated (yellow) and tissue (blue) lung regions (A.b-D.b) are provided for each group of male mice. Representative H&E-stained lung images from TNF-Tg male sedentary versus running mice are shown, where little change in morphology was appreciated in the tissue as a whole or adjacent to the arterioles (black arrows) (E.a-F.b). Black scale bar = 500μm (E,F.a), yellow scale bar = 50μm (E,F.b). Supplementary Table 1. Sample sizes for running data. A table is provided with the sample sizes (number of animals) for each group and timepoint. For each group (WT male/female, TNF-Tg male/female), the sample sizes are shown at the following time points (days, outcomes in 10-day median blocks): 65 / 75 / 85 / 95 / 105 / 115 / 125 / 135. Reductions in sample size indicate mortality during the study period. Supplementary Table 2. Sample sizes for micro-CT outcome measures. A table is provided with the sample sizes for each group and timepoint. The groups include sedentary and running cohorts of WT male/female and TNF-Tg male/female mice. The joint micro-CT outcomes were measured as a change from a 2-month baseline. For the lung and ankle micro-CT, the males show 5 or 5.5-month measurements, while the females exhibit 3 / 4 / 5 or 5.5-month outcomes. The knee micro-CT represents outcomes at 5-month measures for both males and females. Changes in sample size across timepoints either represent exclusion of datasets due to motion artifact or animal mortality during the study period. aFinal timepoint represents 5.5-months of age and experiment unit is number of mice, bfinal timepoint represents 5-months of age and experimental unit is number of limbs, *sedentary measurements are derived from historical data in our previous publication [23]. [file 13075_2022_2985_MOESM1_ESM.pptx]

## Slide 1
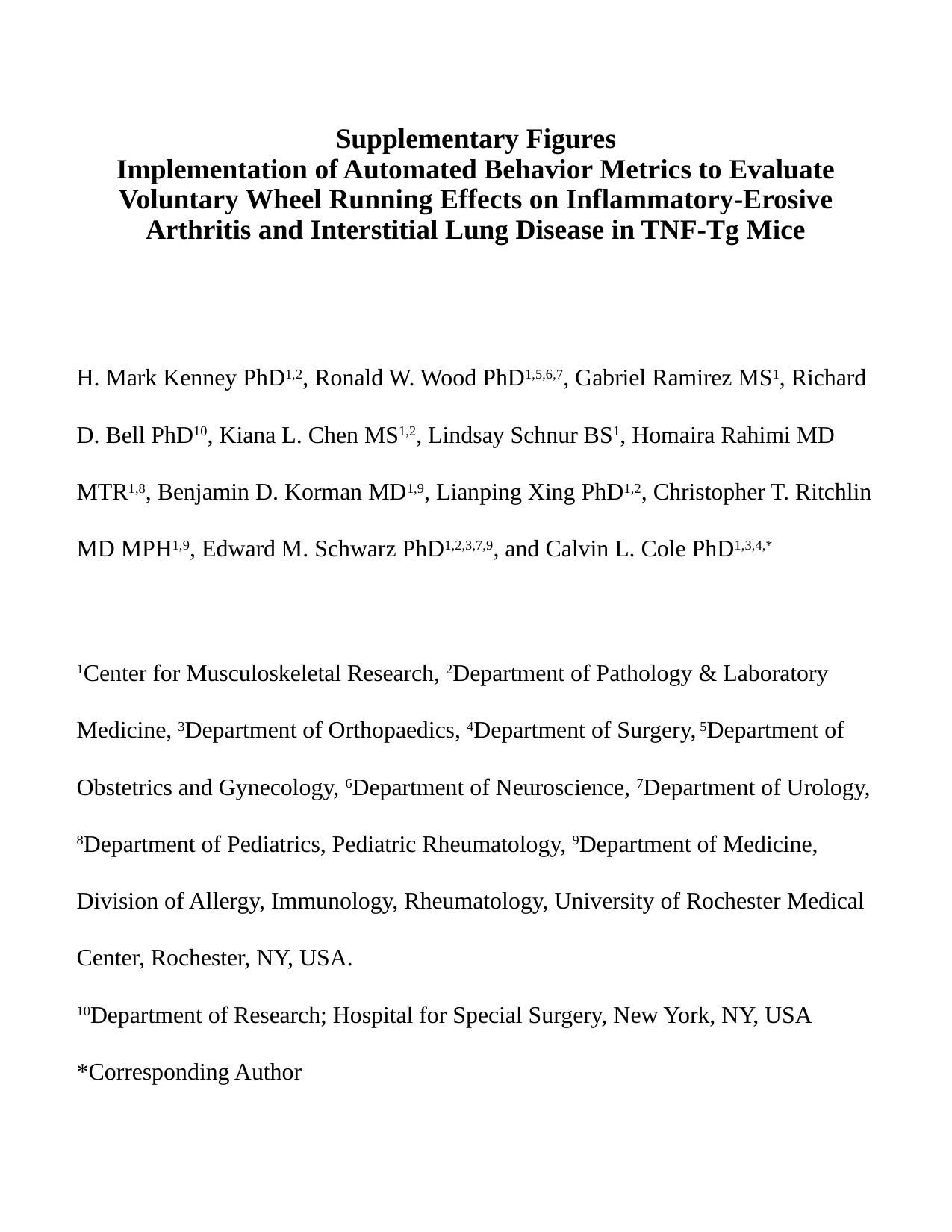

# Supplementary Figures Implementation of Automated Behavior Metrics to Evaluate Voluntary Wheel Running Effects on Inflammatory-Erosive Arthritis and Interstitial Lung Disease in TNF-Tg Mice
H. Mark Kenney PhD1,2, Ronald W. Wood PhD1,5,6,7, Gabriel Ramirez MS1, Richard D. Bell PhD10, Kiana L. Chen MS1,2, Lindsay Schnur BS1, Homaira Rahimi MD MTR1,8, Benjamin D. Korman MD1,9, Lianping Xing PhD1,2, Christopher T. Ritchlin MD MPH1,9, Edward M. Schwarz PhD1,2,3,7,9, and Calvin L. Cole PhD1,3,4,*
1Center for Musculoskeletal Research, 2Department of Pathology & Laboratory Medicine, 3Department of Orthopaedics, 4Department of Surgery, 5Department of Obstetrics and Gynecology, 6Department of Neuroscience, 7Department of Urology, 8Department of Pediatrics, Pediatric Rheumatology, 9Department of Medicine, Division of Allergy, Immunology, Rheumatology, University of Rochester Medical Center, Rochester, NY, USA.
10Department of Research; Hospital for Special Surgery, New York, NY, USA
*Corresponding Author

## Slide 2
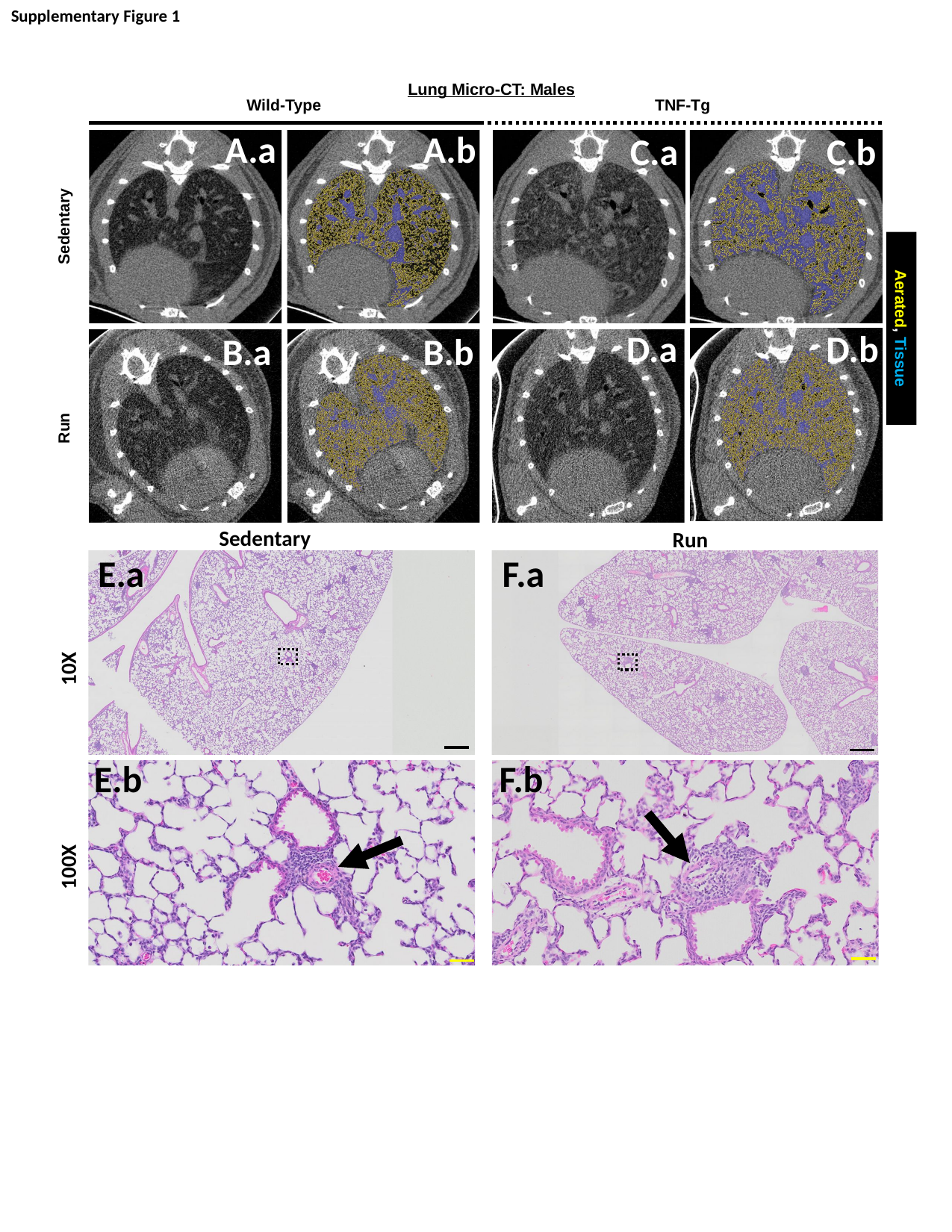

Supplementary Figure 1
Lung Micro-CT: Males
Wild-Type
TNF-Tg
A.b
A.a
C.a
C.b
Sedentary
Aerated, Tissue
D.a
D.b
B.a
B.b
Run
Sedentary
Run
Ε.a
F.a
10X
Ε.b
F.b
100X

## Slide 3
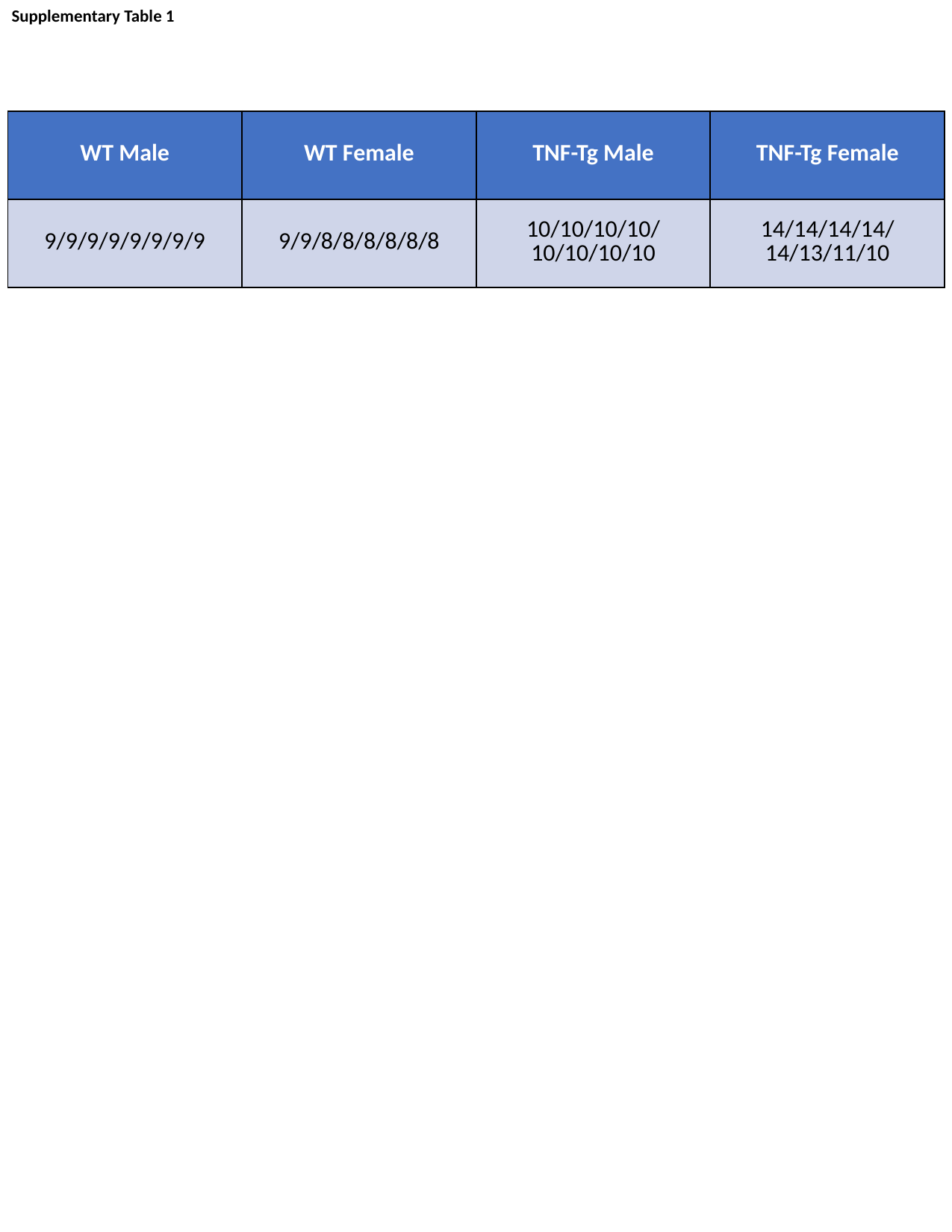

Supplementary Table 1
| WT Male | WT Female | TNF-Tg Male | TNF-Tg Female |
| --- | --- | --- | --- |
| 9/9/9/9/9/9/9/9 | 9/9/8/8/8/8/8/8 | 10/10/10/10/ 10/10/10/10 | 14/14/14/14/ 14/13/11/10 |

## Slide 4
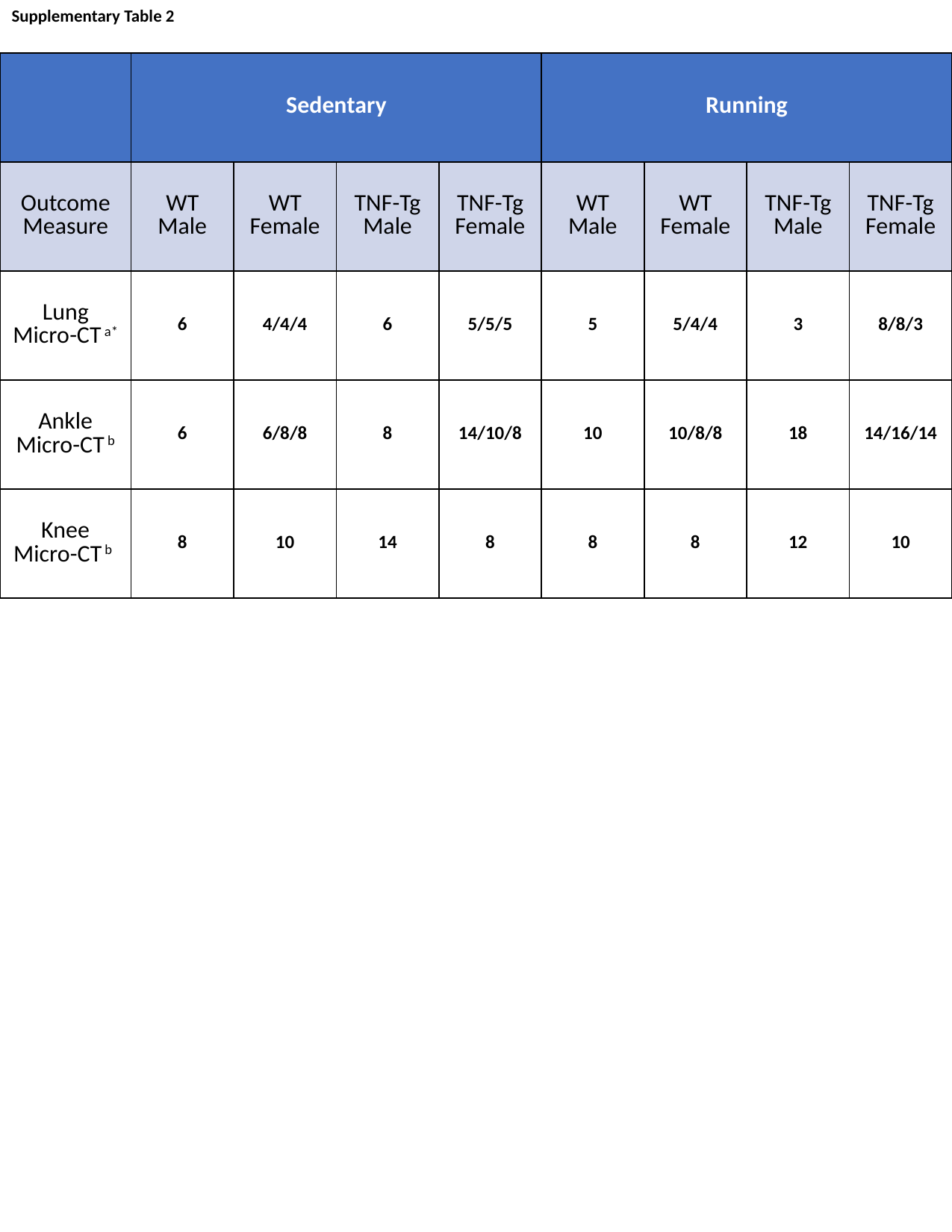

Supplementary Table 2
| | Sedentary | | | | Running | | | |
| --- | --- | --- | --- | --- | --- | --- | --- | --- |
| Outcome Measure | WT Male | WT Female | TNF-Tg Male | TNF-Tg Female | WT Male | WT Female | TNF-Tg Male | TNF-Tg Female |
| Lung Micro-CT a\* | 6 | 4/4/4 | 6 | 5/5/5 | 5 | 5/4/4 | 3 | 8/8/3 |
| Ankle Micro-CT b | 6 | 6/8/8 | 8 | 14/10/8 | 10 | 10/8/8 | 18 | 14/16/14 |
| Knee Micro-CT b | 8 | 10 | 14 | 8 | 8 | 8 | 12 | 10 |
